# Radiologists in the IT era: the Saitama experience

**DOI:** 10.2349/biij.2.3.e41

**Published:** 2006-07-01

**Authors:** S Tsukuda, K Inoue, A Heshiki

**Affiliations:** Department of Radiology, Faculty of Medicine, Saitama Medical University, Moroyama, Japan

**Keywords:** PACS, EMCS, HIS, RIS, Japan

## Abstract

In recent years, intra-hospital computerisation including picture archiving and communication system (PACS) and electronic medical chart system (EMCS) has been rapidly introduced in Japan. The current system has, however, encountered many problems, such as, storage format of images, quality of diagnostic monitors, and compatibility of PACS and EMCS introduced by multi-vendors. In 2003, Saitama Medical University Hospital introduced PACS and EMCS, which can prevent inconsistency and loss of medical care information and can be linked to provide high quality medical care. This paper describes how radiologists should be involved in a hospital information system as specialists of PACS, based on our experience.

## INTRODUCTION

In recent years, there has been rapid intra-hospital computerisation in Japan due to the growth of information technology (IT). Current circumstances are, however, such that while there are highly advanced hospitals, there are also hospitals that rely on conventional manpower. It is believed that IT introduction in medical care, to the extent of what is considered full-scale and practical, has just begun. This paper describes how radiologists should be involved in a hospital information system withina chaotic IT scenario in hospitals and describes problems with the PACS/EMCS currently in operation.

In Japan, the introduction of PACS, which began in the 1980s, initially involved incorporation of a radiology information system (RIS) in a network associated with medical imaging devices, such as, CT and MRI machines. Afterwards, instances of the introduction of PACS in combination with electronic medical charts increased because of the improved connectivity of testing equipment. This improvement was a result of the introduction of DICOM standards and national measures to introduce IT.

By 2001, PACS had been introduced in 1,178 facilities in Japan. The number rapidly increased to 2,342 facilities in 2003 [1]. PACS implementation encountered problems with storage format due to the large amounts of image data produced and the quality of images distributed. Today, a storage format with lossy compression is often chosen because of limitations on storage media, and many facilities distribute reference images to individual departments. In such instances, low-quality images are displayed on low-image-quality terminals. Proceeding with medical care while referring to these images is extremely risky, and it also carries the potential of medically related lawsuits. Currently, however, installing high-image-quality diagnostic monitors in every department is impossible because of the excessive cost involved. Therefore, diagnosing lossless images with high-definition monitors in radiology and ensuring simultaneous distribution of diagnostic reports and reference images is absolutely essential. This will ensure the quality of medical care while minimising unessential network traffic [2].

**Figure 1:**
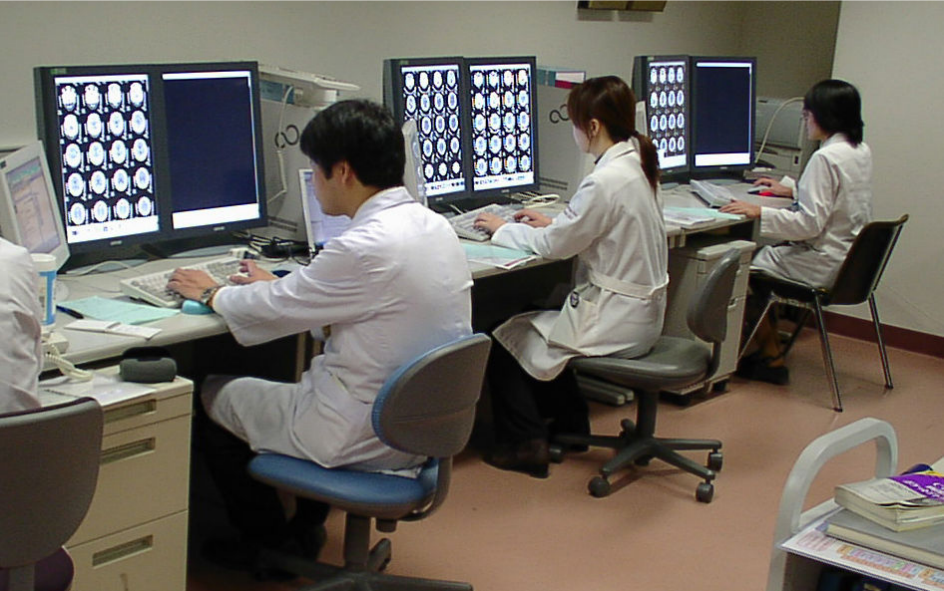
PACS diagnostic workstations at the Saitama Medical University Hospital.

**Figure 2:**
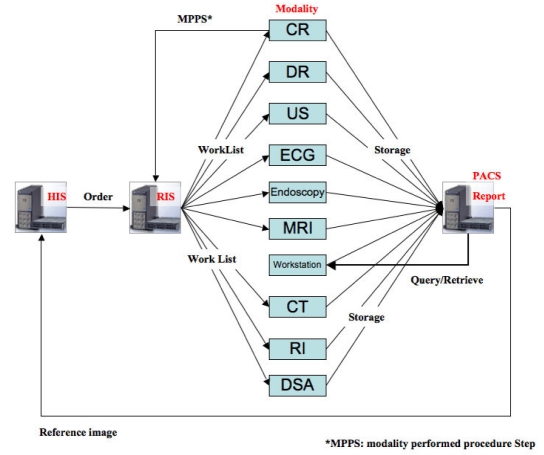
PACS at Saitama Medical University Hospital.

**Figure 3:**
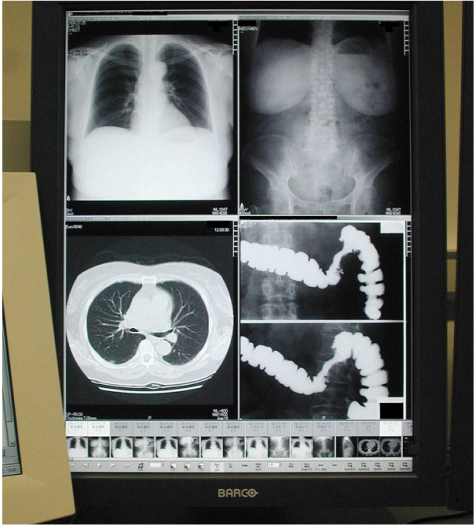
Multimodality images may be displayed simultaneously.

Based on these concepts, PACS was fully introduced at the Saitama Medical University Hospital in 2003 and, subsequently, EMCS was introduced in January 2005 (Figure 1). Therefore, medical images, such as, plain films and results of CT, MRI, ultrasound exams, and endoscopy can be referred to on electronic medical charts (Figure 2). The quality of monitors in electronic chart terminals used by individual departments is not sufficient for diagnosis, thus image assessment based on electronic charts is only for reference. A radiology specialist will then drafts a report based on all of the images produced on a high-quality monitor linked directly by a dedicated line to a server and distributes it to each department within the shortest possible time delay. A clinician lists a patient’s test results with an electronic chart terminal, which then enables immediate reference to images and diagnostic reports. This introduction of EMCS and PACS can prevent the inconsistency and loss of medical care information and can be linked to provide advanced medical care more efficiently. For radiologists, advantages of the introduction of PACS and electronic medical charts are as follows. There is no need to locate films to interpret, remove them from the film jacket, and view them in order on a film viewer as was done before, and the time available for image interpretation has increased [3]. Previous exams and other diagnostic and imaging information required for interpretation can be easily referred to and compared (Figure 3). Easier to understand reports can be provided by attaching diagrams to images. More accurate diagnosis and measurement are possible because of the 3D image processing features of image interpretation terminals. Despite these advantages, however, there are some problems. Electronic medical charts lack the readability of conventional paper medical charts and drop in speed as a result of large amounts of access requests. In addition, keyboard entry takes time, reducing the time spent with the patient and the amount of medical care information listed in the documents, such as, diagnostic referrals. Introduction of faster terminals and networks and extra features for use in keyboard entry and speech-recognition systems is needed.

Considering current conditions from a radiologist’s perspective, images that must be handled by radiologists are increasing exponentially, primarily as a result of the introduction of multidetector row CT. The current shortage of manpower is irrefutable. In this IT era, providing computer-assisted diagnostic applications as a standard on diagnostic terminals is one means of reducing the burden on diagnosticians and ensuring the quality of medical care. In the past, computer-assisted diagnosis was primarily intended for plain films and mammography, but recently, lesion detection in multi-cross-sectional images, such as, CT has also been attempted [4]. These may serve as a means of tackling the increase in image information.

PACS, which is expected to expand further in the future, has a problem with compatibility. Currently, PACS and EMCS introduced by individual medical facilities are multi-vendor, and the reality is that most of the systems are not fully compatible. Radiographic images retain some degree of compatibility and connectivity because of the DICOM [5] format, but information on EMCS is particular to each medical facility. In the future information must, to some extent, be standardised and shared between PACS and EMCS. At the same time, however, the protection of personal information and security of data transmission must not be neglected.

## CONCLUSION

In this IT era, radiologists must play a leading role in the introduction and the implementation of information systems, including PACS. They must also be responsible for the quality of information that flows within the system. In addition, as a part of intra-hospital computerisation, images undiagnosed by a diagnostic imaging specialist must not be distributed to individual departments without being checked by a radiologist. Even in this era of PACS, real-time involvement in medical care is the role of the responsible radiologist. To that end, it is essential to have diagnosticians who can efficiently handle large numbers of images and possess skills to handle 3-dimensional images that will definitely increase in the future.
